# Kernel density estimation of CSD distributions - an application to knowledge based molecular optimisation

**DOI:** 10.1186/1758-2946-6-S1-P10

**Published:** 2014-03-11

**Authors:** Patrick McCabe, Oliver Korb, Jason Cole, Robin Taylor

**Affiliations:** 1 CCDC, 12 Union Road, Cambridge, CB2 1EZ, UK; 2 Taylor Cheminformatics Software, 54 Sherfield Avenue, Rickmansworth, Herts., WD3 1NL, UK

## 

The Cambridge Structural Database ( CSD ) contains a large amount of molecular structure data ( bond length, bong angle and torsion angle data.) Much of this data has previously been extracted in histogram form and provided in the Mogul program. Histograms however have several disadvantages e.g. they are not smooth, they depend on bin widths and bin end points.

Kernel density estimators do not bin data and have no end points but centre a kernel function at each data point and smooth kernel functions will generate smooth density estimates [1]. A difficulty of the approach though is how wide to make the kernel functions.

In this work kernel density estimation is used to generate probability density functions ( pdfs ) for bond length, bond angle and torsion angle histograms derived from the CSD. Gaussian kernels are used for bond length and bond angle data and a von Mises kernel is used for the torsion angle data [2]. The resulting pdfs are smooth and are suitable for application to molecular geometry optimisation.
